# First Case of Sperm Detection by Testicular Sperm Extraction in a Patient With XXY/XX Mosaicism

**DOI:** 10.1210/jcemcr/luaf096

**Published:** 2025-05-15

**Authors:** Natascia Tahani, Francesco Cargnelutti, Matteo Spaziani, Donatella Paoli, Massimiliano Caprio, Andrea Isidori

**Affiliations:** Department of Experimental Medicine, Section of Medical Pathophysiology and Endocrinology, Sapienza University of Rome, Rome 00161, Italy; PhD Programme in Endocrinological Sciences, Sapienza University of Rome, Rome 00161, Italy; Department for the Promotion of Human Sciences and Quality of Life, San Raffaele Roma Open University, Rome 00166, Italy; Department of Experimental Medicine, Section of Medical Pathophysiology and Endocrinology, Sapienza University of Rome, Rome 00161, Italy; Department of Experimental Medicine, Section of Medical Pathophysiology and Endocrinology, Sapienza University of Rome, Rome 00161, Italy; Department of Theoretical and Applied Sciences, eCampus University, Novedrate 22060, Italy; Department of Experimental Medicine, Section of Medical Pathophysiology and Endocrinology, Sapienza University of Rome, Rome 00161, Italy; Laboratory of Seminology—Sperm Bank “Loredana Gandini,” Policlinico Umberto I, Rome 00161, Italy; Department for the Promotion of Human Sciences and Quality of Life, San Raffaele Roma Open University, Rome 00166, Italy; Laboratory of Cardiovascular Endocrinology, IRCCS San Raffaele, Rome 00166, Italy; Department of Experimental Medicine, Section of Medical Pathophysiology and Endocrinology, Sapienza University of Rome, Rome 00161, Italy

**Keywords:** Klinefelter syndrome, XXY/XX mosaicism, spermatogenesis, surgical testicular sperm extraction, case report

## Abstract

Klinefelter syndrome (KS) is a chromosome disorder characterized by small firm testes, gynecomastia, hypogonadism, and abnormally elevated concentrations of follicle-stimulating hormone (FSH). Most KS patients show a classic 47,XXY karyotype, while about 20% have other numeric sex chromosome abnormalities, including mosaicisms. 47,XXY/46,XX mosaicism is extremely rare, and has been reported in just 10 individuals with features suggestive of KS. None of these had any spermatozoa in their ejaculate or testicular samples.

We describe the first case of sperm retrieval in a 19-year-old male patient with a 47,XXY/46,XX chromosomal pattern, assessed on 100 metaphases. Since 2 semen analyses had shown azoospermia, the patient underwent surgical testicular sperm extraction (TESE): Numerous primary spermatocytes, spermatids, and spermatozoa with normal morphology and nonlinear motility were found in the right testis, while few spermatocytes and rare immobile spermatozoa were observed in the left testis. Immediate cryopreservation of the sample was performed in our sperm bank for future use in assisted reproductive technology.

This case underscores the rarity and complexity of this genetic condition and highlights the importance of early fertility assessment and intervention in patients with a 47,XXY/46,XX karyotype.

## Introduction

Klinefelter syndrome (KS) was first described by Harry F. Klinefelter in 1942 as an endocrine disorder characterized by small firm testes, gynecomastia, hypogonadism, and abnormally elevated concentrations of follicle-stimulating hormone (FSH) [1]. It is the most common human sex chromosomal abnormality, with an incidence of 1 in 600 newborn boys [2]. About 80% of KS patients show a classic 47,XXY karyotype, while 20% have higher-grade aneuploidies (48,XXXY, 48,XXYY, 49,XXXXY) or other numeric sex chromosome abnormalities, as 46,XY/47,XXY mosaicism, or structurally abnormal X chromosomes [3, 4].

47,XXY/46,XX mosaicism is extremely rare and has been reported in just 10 cases of individuals with features suggestive of KS [5-14], in 6 cases of ovotesticular disorder of sex development [15-20], in 1 case of ovarian hypoplasia [21], and in 1 patient with a normal female phenotype [22] (Table 1). Testicular biopsy has been performed in only a few cases [8, 10], and no spermatozoa were found in any of them.

**Table 1.** Literature cases of 47,XXY/46,XX mosaicism

Karyotype	First author. Year	Clinical features
XXY/XX	Ford, CE [5]. 1959	Klinefelter syndrome
XXY/XX	Nowakowski, H [6]. 1960	Klinefelter syndrome
XXY/XX	Crooke, AC [7]. 1960	Klinefelter syndrome
XXY/XX	Hecht, F [8]. 1966	Klinefelter syndrome
XXY/XX	Matsuki, S [9]. 1999	Klinefelter syndrome
XXY/XX	Velissariou, V [10]. 2006	Klinefelter syndrome
XXY/XX	Song, JS [11]. 2014	Klinefelter syndrome
XXY/XX	Tachon, G [12]. 2014	Klinefelter syndrome
XXY/XX	Mohd Nor, NS [13]. 2016	Klinefelter syndrome
XXY/XX	Low, KJ [14]. 2017	Klinefelter syndrome
XXY/XX	Turpin, R [15]. 1962	Ovotesticular DSD
XXY/XX	Perez-Palacios, G [16]. 1981	Ovotesticular DSD
XXY/XX	Nihoul-Fekete, C [17]. 1984	Ovotesticular DSD
XXY/XX	Bergmann, M [18]. 1989	Ovotesticular DSD
XXY/XX	Kanaka-Gantenbein, C [19]. 2007	Ovotesticular DSD
XXY/XX	Talreja, SM [20]. 2015	Ovotesticular DSD
XXY/XX	Gagnon, J [21]. 1965	Ovarian hypoplasia
XXY/XX	Hamlett, JD [22]. 1970	Female phenotype

Abbreviation: DSD, disorder of sex development.

## Case Presentation

A 19-year-old male patient came to our observation due to a newly diagnosed left-sided grade 3 varicocele. His height was 190 cm and his weight 66.0 kg. Physical examination revealed bilaterally small testes with a maximum volume of 6 mL each, evaluated with a Prader orchidometer. His motor and mental development were reported as normal during childhood and, at the time of our observation, he was attending the final year of high school. No malformations or gynecomastia were observed and his fat distribution was normal. As part of his clinical care, the patient was advised to undergo testicular color Doppler ultrasonography, sex hormonal analysis, and spermiogram.

## Diagnostic Assessment

Scrotal color Doppler ultrasonography was performed using linear high-frequency transducers (5-12 MHz) and employing the Lambert formula, given its higher accuracy with reduced testicular volumes. It revealed testes in situ, both reduced in size. The right testis measured 32 × 18 × 13 mm (volume 5.4 mL) and the left 24 × 19 × 14 mm (volume 4.6 mL). The echostructure was homogeneous for the right testis and slightly inhomogeneous for the left. The examination confirmed a left-sided grade 3 varicocele.

Blood hormone analysis revealed an FSH level of 10.83 mIU/mL (10.83 IU/L) (reference range, 1.38-9.58 mIU/mL; 1.38-9.58 IU/L) and luteinizing hormone of 10.79 mIU/mL (10.79 IU/L) (reference range, 1.79-8.17 mIU/mL; 1.79-8.17 IU/L). Total testosterone was 15.91 nmol/L (458.9 ng/dL) (reference range, 10.40-38.20 nmol/L; 300-1102 ng/dL) and inhibin B was 51.9 pg/mL (51.9 ng/L) (reference range, 80-380 pg/mL; 80-380 ng/L).

Semen analysis showed azoospermia with normal volume and pH, and no germ cells detected. Thereafter, the entire semen sample was centrifuged at 4000 rpm for 10 minutes and the presence of germ cells was evaluated after May-Grünwald Giemsa staining. As per good clinical practice, the spermiogram was repeated within a few weeks, confirming the diagnosis of azoospermia.

The reduced testicular volumes, increased gonadotropin levels, and azoospermia were suggestive of hypergonadotropic hypogonadism. Therefore, we suggested the patient undergo cytogenetic and molecular analysis and open testicular biopsy for testicular sperm extraction (TESE). For this purpose, a written informed consent was obtained from the patient.

**Figure 1.**
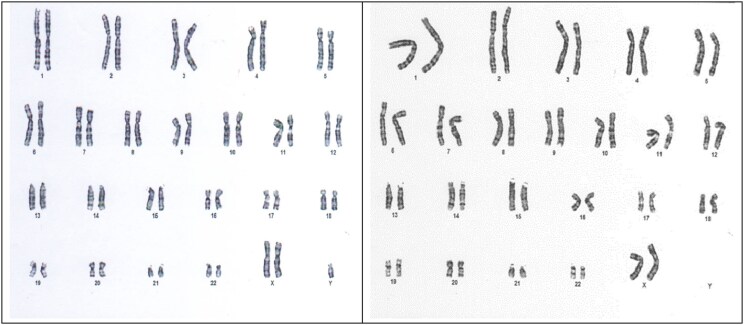
Chromosome analysis showing 47,XXY (92%) and 46,XX (8%) cell lines.

Karyotype analysis was performed on peripheral blood lymphocytes, initially on 30 metaphases and then repeated on 100 metaphases to increase the accuracy of the result. Although the first genetic test showed a classic KS karyotype (47,XXY), the second test on 100 metaphases revealed 2 cell lines: A total of 92% of cells had 2 XX chromosomes and 1 Y chromosome, while 8% had a female sex chromosome complement (46,XX) (Fig. 1).

Microdeletions of the Y chromosome long arm were studied by polymerase chain reaction, performed using specific sequence tagged site primer sets, which spanned the AZFa, AZFb, and AZFc regions of the Y chromosome. No Y chromosome microdeletions were found in our patient.

The molecular analysis of the androgen receptor CAG repeat length showed homozygosity of androgen receptor polymorphism and no abnormalities were observed.

## Treatment

Testicular sperm extraction was assessed by open testicular biopsy (TESE). The testicular tissue was placed immediately into small Petri dishes with sperm-washing medium (Irvine Scientific) and mechanically microdissected with the use of sterile glass microscope slides.

Numerous primary spermatocytes, spermatids, and spermatozoa were found in the testicular tissue of the right testis, with normal morphology and nonlinear motility; in contrast, hypospermatogenesis was observed in the left testis, with few spermatocytes and rare immobile spermatozoa.

Immediate cryopreservation of the sample was performed. Cryopreservation was carried out in nitrogen vapor with rapid freezing, as proposed by Sherman [23]. The sperm suspension was diluted with cryoprotectant medium (Freezing Medium, Test Yolk Buffer, Irvine Scientific) and left to equilibrate at 37 °C for 10 minutes. The suspension was aspirated with a vacuum pump into 500 μL straws, which were sealed and placed in nitrogen vapor for 8 minutes and then immersed in liquid nitrogen at −196 °C.

## Outcome and Follow-up

Six months after the biopsy, laboratory values showed a total testosterone level of 15.4 nmol/L (444.2 ng/dL), luteinizing hormone of 10.95 mIU/mL (10.95 IU/L), and FSH of 10.8 mIU/mL (10.8 IU/L). The patient is not currently taking any endocrine medications.

## Discussion

In this case report, we describe the first case of sperm retrieval in a patient with 47,XXY/46,XX mosaicism, following the detection of sperm in both testes by TESE. Although several individuals with the 47,XXY/46,XX karyotype have been reported in the literature, none of them had sperm in their ejaculate or in testicular samples. For the first time in this particular mosaicism, we found a high number of spermatozoa in testicular samples by TESE and we cryopreserved them in our sperm bank for future use in assisted reproductive technology.

It is well known that men with KS have a low chance of natural paternity, due to a progressive decline in testicular function from puberty onward that seems to be more extensive and precocious in those with higher-grade aneuploidies [4]. A recent study, based on a semi-longitudinal analysis of available cases of individuals with KS, has demonstrated that the gonads of these patients undergo normal development up to pubertal stage G4. It is only thereafter that the reduction in hormones produced by Sertoli and Leydig cells begins, indicating the onset of testicular damage [24]. As the onset of puberty in KS adolescents leads to progressive degeneration of the testicular parenchyma, previous studies have suggested that fertility preservation should be started in adolescents as early as possible [25]. However, current evidence suggests that it should not be offered to patients younger than 16 years because of lower retrieval rates for germ cells by TESE, compared with retrieval rates for individuals between ages 16 and 30 years [26].

Although almost 95% of 47,XXY male patients have no spermatozoa in their ejaculate [27], complete spermatogenesis may be more common in the 47,XXY/46,XY mosaic, and it is associated with more successful reproductive outcomes [3]. However, very little is known about 47,XXY/46,XX mosaicism. This karyotype, in fact, has been reported in only 10 individuals with features suggestive of KS, and whenever testicular biopsy had been performed, no spermatozoa were found.

It is known that the lymphocyte karyotype alone is not predictive of the chromosomal constitution of testicular cells, nor of fertility. Velissariou et al [10] investigated the chromosomal constitution in different tissues from a 47,XXY/46,XX patient and found a high percentage of 46,XX cells in blood lymphocytes and in skin fibroblasts, but exclusively 47,XXY cells in cultured testicular tissue. In the same patient, histologic examination of the testicular tissue showed the presence of hyalinized tubules, fibrotic interstitium, absence of Sertoli cells, and no signs of spermatogenesis.

In our case report, even though the lymphocyte karyotype revealed a 47,XXY/46,XX pattern as seen in the study by Velissariou and colleagues [10], we found some mature sperm cells in both testes. Since 47,XXY germ cells can go through the meiotic and mitotic processes and become mature spermatozoa [28], it may be possible that the spermatozoa we found in the testicular samples originated from 47,XXY germ cells. However, as the chromosomal constitution in the testicular tissue has not been examined for technical reasons, we cannot exclude the existence of 46,XY cells inside the testis, which might be responsible for spermatogenesis. In any case, performing karyotype analysis on as many cells as possible, ideally 100 cells, can improve the sensitivity and reliability of the result, and help to define the likelihood of detecting germ cells that may be exploitable for assisted reproductive technology in KS variants. The variability in sperm retrieval we observed between the two testicles, instead, may be due to several factors, including focal spermatogenesis, asymmetrical testicular degeneration, or sampling variations during TESE.

While testicular volume is generally correlated with spermatogenesis, there is no lower cutoff at which sperm cannot be recovered surgically and, in terms of testicular volume, there is no significant difference between patients from whom sperm can be recovered and those from whom it cannot. Similarly, while FSH and inhibin B levels can be used to determine the mechanism of azoospermia (eg, testicular failure, rather than obstructive azoospermia), neither of these, together or alone, has consistently been shown to predict the chance of sperm recovery on surgical sperm retrieval in KS [29]. Therefore, it could be useful for future studies to focus on identifying markers to determine which men could be expected to have sperm at the time of surgical sperm retrieval.

In conclusion, this case highlights the rarity and complexity of the XXY/XX mosaicism and underscores that not only patients with classic KS, but also those with the XXY/XX karyotype, should undergo semen analysis and testicular sperm extraction in cases of azoospermia confirmed twice. In these patients, fertility assessment should be encouraged at a young age, before germinal epithelium degeneration and seminiferous tubule hyalinization, to increase their chance of fatherhood.

## Learning Points

- The 47,XXY/46,XX mosaicism is an extremely rare condition that can manifest with a variety of phenotypic features.
- In patients with a male phenotype, fertility assessment should be encouraged at a young age to increase their chance of fatherhood.
- Since mature sperm cells could be found in testicular samples, testicular biopsy for sperm extraction should be considered in cases of azoospermia.

## Contributors

All authors made individual contributions to authorship. N.T., F.C., M.S., and A.M.I. were involved in the management of the patient. N.T. and F.C. wrote the first draft of the manuscript. D.P. performed molecular studies, semen analysis, and the cryopreservation of the sample. M.S., M.C., and A.M.I. contributed to writing the final draft. N.T. and M.C. revised the manuscript according to reviewers’ comments. All authors reviewed and approved the final version of the manuscript.

## Data Availability

Data sharing is not applicable to this article as no data sets were generated or analyzed during the current study.

## Abbreviations

FSH: follicle-stimulating hormone
KS: Klinefelter syndrome
TESE: testicular sperm extraction
